# Recognition of small numbers in subset knowers Cardinal knowledge in early childhood

**DOI:** 10.1098/rsos.230474

**Published:** 2023-10-25

**Authors:** Anton Gerbrand, Gustaf Gredebäck, Marcus Lindskog

**Affiliations:** Uppsala Child and Babylab, Uppsala Universitet, Department of psychology, Sweden

**Keywords:** knower level, cardinal recognition, cardinality, understanding number words, eye-tracking

## Abstract

Previous research suggests that subset-knowers have an approximate understanding of small numbers. However, it is still unclear exactly what subset-knowers understand about small numbers. To investigate this further, we tested 133 participants, ages 2.6–4 years, on a newly developed eye-tracking task targeting cardinal recognition. Participants were presented with two sets differing in cardinality (1–4 items) and asked to find a specific cardinality. Our main finding showed that on a group level, subset-knowers could identify all presented targets at rates above chance, further supporting that subset-knowers understand several of the basic principles of small numbers. Exploratory analyses tentatively suggest that 1-knowers could identify the targets 1 and 2, but struggled when the target was 3 and 4, whereas 2-knowers and above could identify all targets at rates above chance. This might tentatively suggest that subset-knowers have an approximate understanding of numbers that is just (i.e. +1) above their current knower level. We discuss the implications of these results at length.

## 1. Introduction

Being able to understand numbers and what they represent is an important part of school readiness [1], academic achievement [2] and success in several areas of adult life (e.g. health self-management; [3]). Thus, the importance of numerical understanding and tools to foster it in childhood cannot be overstated. At the same time, it is unclear and debated exactly how children develop the ability to understand numbers [4–7]. Recent research indicates that *recognizing* cardinalities might be an important aspect of number word understanding (e.g. [8,9]). Two fundamental keys to understanding numbers involve the realization that a number word denotes a specific cardinality of a given set, so that ‘two' refers to exactly two objects, and that number words are mutually exclusive, so that ‘two' cannot be used to specify the cardinality of a set containing more or less than 2 (for further information on number word understanding, see [4,6,10]). One task typically used to explore children's ability to *produce* a given cardinality is the give-n, but how performance on this task relates to the ability to *recognize* cardinality is less investigated [5,11–13].

In the give-n task, children are asked to give a certain number of objects to an interaction partner. As such, this task requires children to produce sets that match specific number words (e.g. in response to the request, ‘Can you give me two?'). Results from studies using this task suggest that children pass through three distinct phases in acquiring number word understanding [14–16]. They begin as pre-knowers who can recite the counting list (1, 2, 3, etc.) but cannot provide the right number of objects when asked, suggesting that they have yet to associate number words with their respective cardinal meaning. Next, children enter the subset-knower phase and can produce some but not all numbers when asked. For instance, a 2-knower can give sets corresponding to the words ‘one' and ‘two' but not to ‘three' or higher (i.e. unknown number words). Theory and current empirical findings suggest that subset-knowers learn number words in a progressive manner [5,11], seeming to first grasp ‘one', followed by ‘two', then ‘three', and so on, until they finally transition into the ‘cardinal principle-knower phase' (CP-knower). At this point, children can produce the corresponding cardinality for all number words they know [15–18].

While the give-n task is an efficient way of measuring children's ability to produce the cardinal sets of number words, it might be less sensitive in assessing *cardinal recognition*—that is, the ability to recognize a given set (e.g. [4–7,19]). First, recent empirical work shows that both visuospatial working memory and expressive vocabulary predict give-n performance among children in longitudinal studies [20], suggesting that the give-n tap into several cognitive processes beyond recognition. Furthermore, Wagner *et al.* [13] show that before subset-knowers learn the exact meaning of small number words (i.e. 1–4), they can still create sets of that size more often than predicted by chance. Furthermore, children that can provide sets above their current knower level (i.e. being able to give *N* + 1) have a higher probability of transitioning into the next knower level in the near future [8]. For example, a 2-knower who gives three when asked to give 1 more than 2 (but also give 3 when asked for other numbers, such as ‘four'), is more likely to become a 3-knower in the next few weeks, compared to a 2-knower that does not demonstrate such an approximate understanding. Together, this indicates that children might recognize some cardinal property of number words before they can produce that specific cardinality reliably (also see [21]), and they might use this approximate understanding to develop a more exact understanding of number words [8].

There is some conflicting evidence regarding cardinal recognition ability within subset-knowers. Using a point-to-X task, tasking children to identify a target cardinality from two different sets (e.g. ‘which has three?'), Silver *et al.* [9] found that 1-knowers could recognize and identify the correct target when the numbers ranged from 1–4. This indicates that the 1-knowers in the sample could recognize and contrast given sets beyond their current knower level. At the same time, Pinhas *et al*. [22] documented a neural marker of surprise in 3–5.5-year-olds that occurred when they were presented with a mismatch between a verbal numerical label (e.g. ‘four’) and a set of objects being presented on a display (e.g. 2). Of note, the surprise reaction was absent in 1-knowers, even when the mismatch trial contained the word ‘one’, indicating no cardinal recognition in 1-knowers. A potential explanation to these diverging results is task design difference. First, comparing a neural marker of surprise with that of a behavioural measurement can be difficult—perhaps the difference in results are due to different outcome measurements. Second, the point-to-X task typically has 12–16 trials (e.g. [8,9,23]), whereas Pinhas *et al*. [22] had 48 trials. Moreover, in Silver *et al*.'s [9] study, if the child did not respond, pointed at the two sets simultaneously, or wanted to change his/her mind, they got a second prompt—with no clear time limit to give their response. This might affect the performance of subset knowers, and has a risk of overestimating children's cardinal recognition.

To further investigate the cardinal recognition ability of subset-knowers, we developed a new eye-tracking task. This task presents a two-forced choice, like that in point-to-X, but differs in some important aspects. First, it allows us to present the participants with more trials (48 trials in total), control for the fact that the target number word is presented an equal number of times (e.g. 1 is the target for 12 trials, 2 is the target for 12 trials, etc.), and show all possible combinations (e.g. 1 versus 2, 1 versus 3, 2 versus 3, etc.) an equal number of times. This should aid in capturing a reliable performance of child participants. Second, it allows us to control for the fact that each child has the same amount of time to respond, and making sure that each child only gets the target prompt once—meaning the conditions are more similar for each participant. Finally, it allows us to measure first gaze. If participants recognized what the target word referenced, they should be expected to look towards the matching target first. This makes it possible to capture a more spontaneous response of cardinal recognition than in that of point-to-X, which we argue should capture children's (approximate) knowledge more readily. With this task in mind, our research question was posed as: do subset-knowers demonstrate an approximate knowledge of small number words? Specifically, can subset-knowers spontaneously recognize the correct cardinal target in a two-forced choice task?

## 2. Methods

### 2.1. Participants

The final sample consisted of 133 participants (mostly white middle class academic parents from [blinded for review]) between 2.6 and 4 years of age (*M*_age_ = 3. 4 years, s.d._age_ = 0.6, min = 2. 6, max = 4. 7; 55% female). To ensure a wide range of knower levels (as determined by the give-n task), we collected data in two waves. The final first wave sample included 40 4-year-old children, see electronic supplementary material for further information on this wave. A large portion of these participants were categorized as CP-knowers (67% according to the give-n task). The second wave included 93 2.5–3.5-year-old children (for power calculations, see electronic supplementary material). Here, most (75%) were pre-knowers, 1-knowers or 2-knowers. For the main analysis, data from both waves were aggregated into one large dataset (for details regarding each wave, see electronic supplementary material). An additional five participants were tested and excluded from the analyses because they did not provide data on either task (due to inattentiveness or unwillingness to do the tasks; one participant from the first wave). Parents provided written and verbal consent prior to participation. The study was approved by the regional ethics committee (2019-04305).

### 2.2. Design and materials

To investigate how cardinal recognition relates to cardinal production performance, we developed an eye-tracking task (using a Tobii TX300; 60 Hz; Tobii Technology, Stockholm, Sweden) that assessed children's ability to recognize cardinalities. Children sat on their parent's lap at approximately 60 cm of viewing distance from a 23-inch monitor. Before data collection, we ran a standard 5-point calibration procedure [24], which was repeated until results were satisfactory. Parents were instructed to act as neutral as possible, and not comment or help children's performance. Only in a few instances did parents not act according to instructions, which also happened to be the children who were also excluded due to inattentiveness or unwillingness to do the task (i.e. five participants, see data reduction).

**Figure 1.**
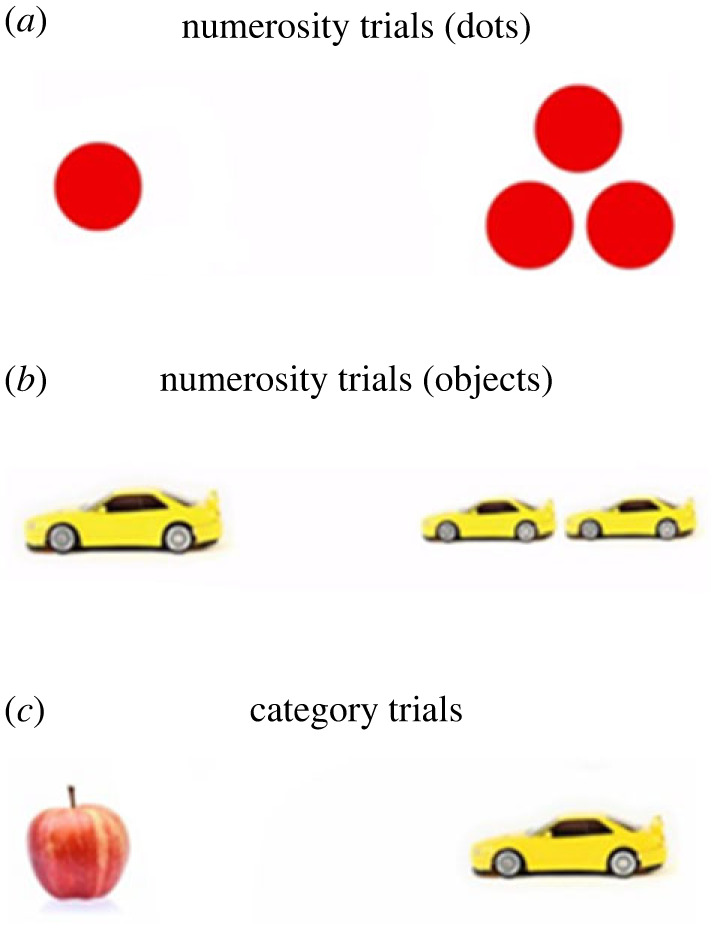
Snapshots of the stimuli used in each trial. (*a*) Illustration of the numerosity trials, presented with dots (same item size). (*b*) Illustration of the numerosity trials, presented with everyday objects (same overall area). (*c*) Illustration of the category trials, presented with everyday objects. Note that in the actual stimuli presentation, all sets of objects had the same distance between them.

During numerosity trials, participants were presented with two sets of objects consisting of either dots (blue, red, purple or green) or objects (balls, cars, bananas or apples), differing in cardinality (1–4 objects in each group). Each colour of the dots, and each object, were presented a total of 6 times as the target (including category trials, see below). We chose to present numerosity trials as either dots or objects, as to be able to investigate a secondary question of if stimuli familiarity affected performance. See electronic supplementary material for further information and analyses. We chose to only present participants with the numbers 1–4 for two reasons; (1) most importantly, it is unclear what children at each subset-knower level understand about small numbers (i.e. 1–4; [9,22]), and (2) since this is a newly developed eye-tracking task, introducing a large range of numbers could make the evaluation of the task and interpretation of performance difficult (also see [13] for evidence that subset-knowers do not understand large numbers). During the first 5 s of each trial, an image of the two sets was presented, and a voice asked participants to find the set with a particular cardinality (e.g. ‘Look [‘dots' or ‘cars']! Can you find [‘one', ‘two', ‘three' or ‘four’]?'). The array of sets always consisted of identical objects. All prompts were presented in Swedish. The image stayed on the screen for another 5 s after the prompt. To ensure task integrity, participants were also presented with category trials with two different objects (balls, bananas, cars or apples) and then asked to find one of them (e.g. ‘Look! Can you find the car?'). All other aspects of these trials were identical to the numerosity trials described above (figure 1).

Each session included 48 numerosity trials and 24 category trials, separated into three blocks. Each block consisted of 16 numerosity trials (50% object trials, 50% dot trials) and 8 category trials, with brief attention-grabbing stimuli between each. Each block lasted approximately 6 min. The positions of the target and distractor were counterbalanced over blocks and trials. The order of trials was also counterbalanced so that the same type of trial did not occur twice in a row. In addition, the order of trials was counterbalanced over blocks. To ensure that participants did not use other visual cues to find cardinality, 50% of trials equated the average object size, so that all objects within a category had identical area (dots, 2.4 × 2.3 visual degrees; objects, 2.3 × 2.4 visual degrees). The remaining 50% of trials equated cumulative area, so that each set of objects had an identical area (dots, 4.1 × 1.9 visual degrees; objects, 4.4 × 1.9 visual degrees).

Participants took a short break (approx. 4 min) between the first and second blocks to reduce fatigue and maintain attention to the stimuli. Between the second and third blocks, participants performed the give-n task [16], with a few minutes break between the tasks, and parents filled out the Home Numeracy Scale questionnaire (see electronic supplementary material for further information; [1,25]).

At the start of the give-n [16], the experimenter explained that children were going to give pearls to a stuffed animal. The experimenter presented 12 pearls and a plate, and informed the participants that they should put the requested number of pearls on the plate and give it to the experimenter to pass on to the stuffed animal. For each trial, participants were asked to provide a number of pearls: ‘Can you give n [1, 2, 3, or 4]?' When the child had placed as many pearls as they wished on the plate, the experimenter took the plate and placed it in front of the stuffed animal. At this point, the experimenter asked, ‘Is that n [1, 2, 3, or 4]?' If the child said ‘yes', the experimenter moved on to the next trial. If the child said ‘no', the experimenter said, ‘You can change so that it is n [1, 2, 3, or 4] on the plate'. If the child did not change the number of pearls, the experimenter moved on to the next trial. If the participant changed the number of pearls, the experimenter asked the same question again. Regardless of a yes or no response, the experimenter went on to the next trial.

Participants were presented with 12 trials in a fixed order, a choice we made for two reasons. First, we wanted to ensure that for each participant, the experience of the task was as similar to possible. Second, a fixed order is appropriate when calculating the probability of a participant's knower level (see data reduction; [26]). The order was counterbalanced such that numbers were *never* presented in the natural order (i.e. 1–4), and that the same number did not occur twice in a row (the order of presentation was: 2, 1, 4, 3, 1, 3, 2, 4, 2, 4, 3, 1). The task lasted for approximately 10 min.

### 2.3. Data reduction

The eye-tracking data was exported from Tobii studio (Tobii Technology, Sweden) to TimeStudio [27], software operating within the MATLAB environment (Version 9.3.0.7; The Mathworks, Natick, MA). Within TimeStudio, we defined areas of interest covering the two sets of objects and the left or right side of the screen at 18.82 × 26.37 visual degrees, and the exclusion criterion of less than 20% looking time towards the screen. We reasoned that this criterion would remove any spurious first gaze data (see dependent measure below), and its application (in combination with exclusion due to inattentiveness or lack of valid data) resulted in exclusion of 48% of all trials. On average, participants contributed 29.7 trials (s.d. = 12, Min = 12, Max = 65). We note that a relatively high attrition is common in eye-tracking research with children (e.g. [28]).

We operationalized recognition as a proportional score of first gaze towards the target. The score was calculated as follows: FG _target_/(FG_target_ + FG_distractor_). To avoid analysing the first gaze before the participants had the chance to process the target word prompt (number of words in numerosity trials and nouns in category trials), we analysed first gaze 200 ms after the prompt ended (accounting for the reaction time of the oculomotor system; [29]). The proportional score ranged from 0 to 1 (first gaze directed to the target = 1, at random = 0.5, first gaze directed to the distractor = 0). Thus, a recognition score higher than 0.5 would suggest that participants looked towards the target first. Seven participants were excluded from this task, due to not following instructions/not wanting to do the task (5), or because they had no valid first gaze in any trial (2). The excluded participants did not differ in any key variables (e.g. knower level), compared to the retained participants. We also analysed the proportional looking time to the target area of interest during the 5 s after the end of the verbal cue. This measure and the results from this analysis are reported in the electronic supplementary material because they are highly similar to those described in the main text.

The give-n task was coded in accordance with Negen *et al*. [26], using a model to calculate the probability of a child's knower-level based on task performance. The model weighed in both a flat prior and posterior probability of the child's knower level, so that before the task started, each knower level was equally likely. The child's response affected the posterior probability, such that a child responded with 2 objects when asked for 2, the likelihood of the child being a 2-knower increased, while the likelihood of being any other ‘type’ of knower decreased. Eight participants were excluded from the give-n task because of inattentiveness to the task or procedure (6) or because they did not want to do the task (2).

To answer our research question, we performed two main analysis blocks, followed by exploratory analyses. The first analysis block consisted of single-sample *t*-tests assessing if cardinal recognition performance exceeded chance levels (i.e. H_0_ = 0.5). We performed this analysis within category trials to evaluate if we could conceptually replicate the findings of Bergelson & Swingley [30], and for numerosity trials in order to start investigating our main research question.

The second analysis block included Spearman correlations to investigate potential multicolinearity and a series of stepwise mixed models. In each mixed model, we imputed missing data using Maximum-Likelihood estimation, which estimates missing data based on the available observed data [31]. Table S1 in electronic supplementary material for information on the number of valid trials used in the models. **Mixed model 1** included cardinal recognition (proportional first gaze) as the dependent variable, participant as a random effect, and knower level and target cardinality as fixed effects (age was removed because of multicolinearity; see Results). This approach allowed us to investigate the relationship between knower level and cardinal recognition. Model 1 demonstrated a main effect of knower level, so we also performed a post-hoc multiple comparison test. The post-hoc analyses suggested a potential difference between 1-knowers and 2-knowers. To investigate this further, we performed exploratory analyses. Specifically, we split the dataset into two groups based on knower level and then ran model 1 again on each respective group. Thus, the **mixed model 1.1** was performed with only pre- and 1-knowers, and **mixed model 1.2** was performed with only 1- and 2-knowers (also see mixed model 1.3 in electronic supplementary material where we explore potential differences between 2- and CP-knowers).

As mixed model 1 and subsequent model 1.2 suggested that 2-knowers were better at identifying 3 and 4 compared to 1-knowers, we wanted to investigate whether this performance was due to 2-knowers having more known number words to contrast unknown numbers or not. Therefore, **mixed model 2** aimed to investigate whether 2-knowers had equal performance in trials presenting sets of 2 versus 3 compared to trials presenting 3 versus 4. The rationale here is that if 2-knowers show similar performance in both combinations, it would suggest that they can recognize sets of three and four even without a known set (e.g. 2) to contrast it with.

To ensure the reliability of these mixed models, we also performed a series of follow-up models and assumption checks, controlling for stimuli type (dots versus object trials) and experience of numbers in the home, and included proportional looking time rather than first gaze as dependent variable. These analyses are described and reported in the electronic supplementary material. All data and analyses can be accessed at OSF [https://osf.io/k3bzq/?view_only=ee8417ffe794478886bccf96f64f4d38].

## 3. Results

**Figure 2.**
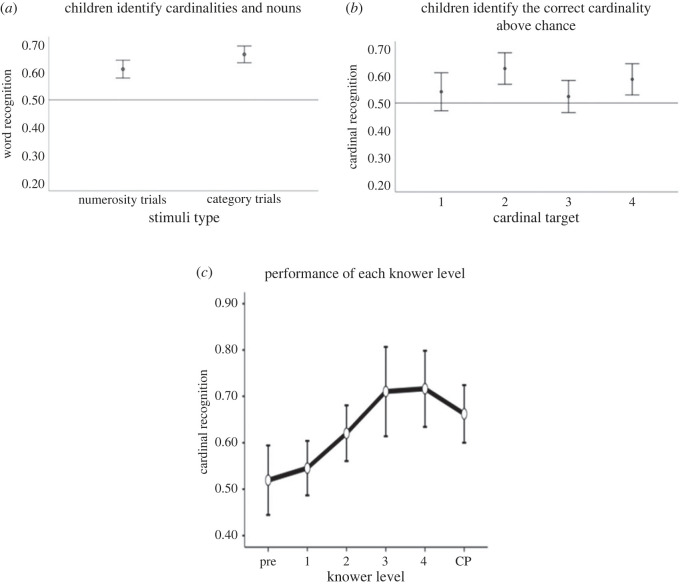
Illustrations of cardinal recognition performance on a group level (2*a* and 2*b*), and across all knower levels (2*c*), respectively. Note that for 2*a* and 2*b*, all performance is above chance (i.e. *p* < 0.05).

Results of single-sample *t*-tests (H_0_ = 0.5) demonstrated that the children performed above chance during numerosity trials (*M* = 0.61, s.d. = 0.19, *t*_130_ = 6.8, *p* < 0.001), looking at the target more frequently than the distractor across all cardinalities (‘one', *M* = 0.56, s.d. = 0.33, *t*_126_ = 2.4, *p* = 0.02; ‘two', *M* = 0.65, s.d. = 0.28, *t*_125_ = 5.8, *p* < 0.001; ‘three', *M* = 0.57, s.d. = 0.30, *t*_128_ = 2.7, *p* = 0.008; and ‘four', *M* = 0.62, s.d. = 0.29, *t*_127_ = 4.6, *p* < 0.001). Participants also performed above chance in category trials (*M* = 0.66, s.d. = 0.18, *t*_130_ = 10.7, *p* < 0.001), conceptually replicating the category word recognition phenomenon reported in Bergelson & Swingley [30] with our task and dependent measure (figure 2*a,b* for illustrations). Next, we investigated whether knower level was related to performance in the eye-tracking task (figure 2*c*). See table 1 for frequency of the different knower levels in the sample.

**Table 1.** Frequency of the different knower levels (KN level) in the sample. Note. For knower level, *n* = 125.

KN level	pre-knower	1-knower	2-knower	3-knower	4-knower	CP-knower
frequency	18	30	27	10	7	33

### 3.1. Model 1. Does knower level relate to the ability to recognize cardinality?

Spearman correlations demonstrated a strong association between knower-level and age (*r*_124_ = 0.71, *p* < 0.001; see electronic supplementary material for full correlation matrix). As such, we excluded age from the model. Thus, the final model included cardinal recognition as the dependent variable, with a random intercept for participants, and fixed effects for knower level and target cardinality. The model demonstrated that both knower level (*F*_5,138_ = 4.70, *p* < 0.001) and target cardinality (*F*_3,779_ = 3.02, *p* = 0.03) affected performance, but there were no significant interaction effects^1^. For illustrations of knower level performance, figure 2*c*. Post-hoc comparison analysis was performed to further evaluate the effect of cardinal target and knower level. For cardinal target, post-hoc analyses demonstrated a significant difference when the target was 2 compared to when the target was 3, and when the target was 1 compared to when the target was 2 (table S4 in electronic supplementary material for the complete comparison). To investigate this further, we also examined whether performance in the different combinations of numbers (e.g. 1 versus 2, 1 versus 3, etc.) might drive this effect. Here, we saw that participants performed somewhat worse in the combination 2 versus 3, and 3 versus 4, respectively (but still at rates above chance; table S5 in electronic supplementary material for the performance across all number combinations). This could suggest that it was relatively difficult to identify the target 3 in two of the possible three combinations it was presented in. Consequently, it might explain why participants identified the target 2 more often than the target 3. However, this cannot explain why there is a difference between target 1 and 2, or why we saw no other differences between the cardinal targets. Thus, at this group level, with all knower levels taken into account, it is difficult to assess what drives this effect.

For the main effect of knower level, post-hoc comparisons demonstrated significant differences between pre-knowers and 2-, 3-, 4- and CP-knowers. Furthermore, there was a difference between 1-knowers and 3-, 4- and CP-knowers. Table 2 lists significant differences (table S3 in electronic supplementary material for the complete comparison table). To gain a better understanding of what these comparisons might reflect, we chose to descriptively examine each knower level's performance on each target (table 3). Looking at the table, it seems that pre-knowers perform at random for all targets, as expected. Interestingly, it seems that 1-knowers perform above chance rates when the target is 1 and 2, but at random for all other targets. Looking at 2-knowers, it seems that they performed above chance rates for all targets (similar to 3- to CP-knowers). Thus, it seems that 1-knowers perform better than pre-knowers when the target is 1 and 2, and worse than 2-knowers (and equal to pre-knowers) when the target is 3 and 4.

**Table 2.** All significant post-hoc comparisons between different knower levels, with cardinal recognition as dependent variable. Note. 0 = pre-knower, 1 = 1-knower, 2 = 2-knower, etc. and 5 = cardinal principle-knower.

post-hoc comparisons—knower level							
comparison							
knower level		knower level	difference	s.e.	*t*	d.f.	*p*
0	—	2	−0.10114	0.0496	−2.0377	132	0.044
0	—	3	−0.19109	0.0633	−3.0185	176	0.003
0	—	4	−0.19702	0.0576	−3.4228	156	<0.001
0	—	5	−0.14284	0.0503	−2.8386	134	0.005
1	—	3	−0.16500	0.0583	−2.8301	198	0.005
1	—	4	−0.17093	0.0523	−3.2687	166	0.001
1	—	5	−0.11674	0.0442	−2.6409	138	0.009

**Table 3.** Mean performance (of cardinal recognition), and 95% confidence intervals for each target, per each knower level*.*

cardinal target				
knower level	1	2	3	4
Pre	*M* = 0. 48, CI = 0.35–0.61	*M* = 0.56, CI = 0.42–0.70	*M* = 0.50, CI = 0.38–0.62	*M* = 0.53, CI = 0.39–0.66
1	*M* = 0.58, CI = 0.45–0.70	*M* = 0.63, CI = 0.53–0.73	*M* = 0.44, CI = 0.34–0.54	*M* = 0.52, CI = 0.42–0.63
2	*M* = 0.60, CI = 0.50–0.71	*M* = 0.68, CI = 0.57–0.77	*M* = 0.59, CI = 0.49–0.70	*M* = 0.62, CI = 0.53–0.72
3	*M* = 0.60, CI = 0.41–0.87	*M* = 0.82, CI = 0.70–0.95	*M* = 0.64, CI = 0.41–0.87	*M* = 0.74, CI = 0.61–0.88
4	*M* = 0.69, CI = 0.54–0.85	*M* = 0.72, CI = 0.59–0.86	*M* = 0.64, CI = 0.50–0.78	*M* = 0.82, CI = 0.71–0.92
CP	*M* = 0.63, CI = 0.53–0.72	*M* = 0.66, CI = 0.56–0.75	*M* = 0.72, CI = 0.64–0.79	*M* = 0.66, CI = 0.57–0.76

To explore this pattern further, we first conducted an exploratory mixed model 1.1, where we split the dataset to only include pre- and 1-knowers, repeating the analysis above for this dataset. Next, we conducted a mixed model 1.2, where we split the dataset to include only 1- and 2-knowers, again repeating the analysis for this dataset (also see electronic supplementary material for mixed model 1.3, where we investigate 2-knowers in relation to 3- to CP-knowers further).

#### 3.1.1. Is there a difference in performance between pre- and 1-knowers?

**Figure 3.**
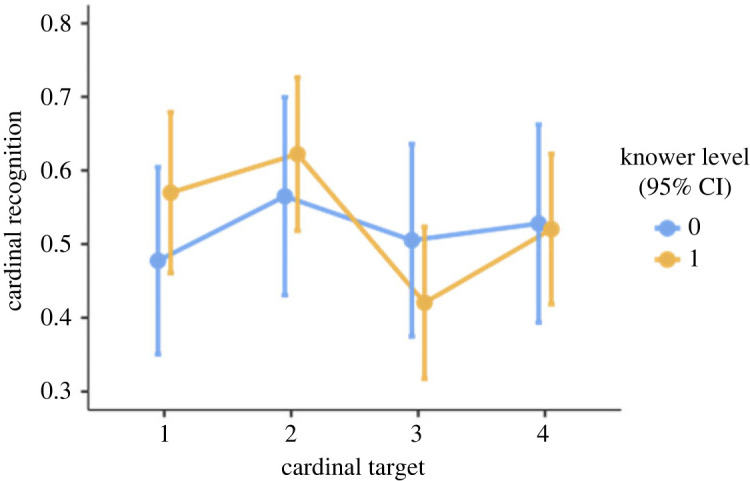
Illustration of cardinal recognition performance for each target, by knower level (0 = pre-knowers, 1 = one-knowers). Error bars denote confidence intervals (95%).

For this exploratory analysis, we split the dataset and included only pre-knowers and 1-knowers, which resulted in 48 participants (18 pre-knowers). The model included cardinal recognition as dependent variable, with a random intercept for participants, and fixed effects for knower level and target cardinality. The mixed model did not demonstrate an effect of knower level (*F*_1, 42_ = 0.09, *p* = 0.77) or target cardinality (*F*_3, 293_ = 1.74, *p* = 0.16), and there were no interaction effects. Thus, this model further suggests that 1-knowers did not perform differently than pre-knowers. However, looking at figure 3, we can again see a descriptive mean difference between the groups for when the target is 1 and 2 (although overlapping), and much more similar performance when the target is 3 and 4. Thus, descriptively, it seems that pre-knowers struggle to find all targets, while 1-knowers struggle to find the target when it is 3 and 4. Perhaps our models cannot find a difference because pre- and 1-knowers are so similar in performance when the target is 3 and 4 (however, performance when target is 1 and 2 is also overlapping, so we should be careful with this interpretation). In any case, it is possible we would find a difference if we had greater statistical power.

#### 3.1.2. Is there a difference in performance between 1- and 2-knowers?

**Figure 4.**
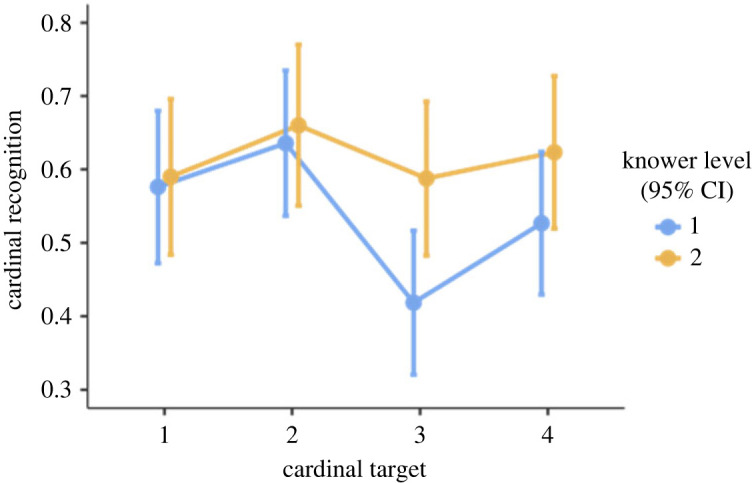
Illustration of cardinal recognition performance for each target, by knower level (1 = one-knowers, 2 = two-knowers). Error bars denote confidence intervals (95%).

For this analysis, we split the dataset and included only 1-knowers (*n* = 30) and 2-knowers (*n* = 27). The model included cardinal recognition as the dependent variable, with a random intercept for participants, and fixed effects for knower level and target cardinality. The mixed model demonstrated an effect of cardinal target (*F*_3, 353_ = 2.73, *p* = 0.04), but not of knower level (*F*_1, 52.8_ = 3.60, *p* = 0.06), and there was no significant interaction effect. Thus, we do not find any significant difference between 1- and 2-knowers; rather, it seems both groups' performances are affected by what the target is. However, looking at figure 4, we can see a descriptive mean difference for when the target is 3 and 4—again indicating that 1-knowers struggle to identify these targets, while 2-knowers do not, at least not as much as 1-knowers. It could suggest that we would find a significant difference between 1- and 2-knowers if we had greater statistical power.

A potential explanation as to why 2-knowers seem to be better at identifying 3 and 4 is that they have an exact understanding of both 1 and 2. This could mean that they can contrast unknown number words (i.e. 3 and 4) to known number words (i.e. 1 and 2) more often than 1-knowers. To investigate this possibility further, we conducted a second mixed model, to see if 2-knowers perform worse in trials when the combination was 3 versus 4, compared to 2 versus 3. If we find a difference here, it would suggest that 2-knowers cannot find 3 and 4 without a known number word to contrast it to.

### 3.2. Can 2-knowers identify the correct target when presented with only unknown sets?

**Figure 5.**
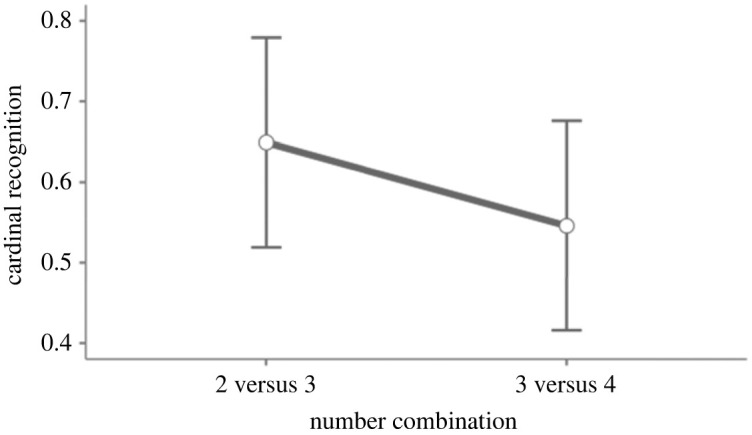
Illustration of cardinal recognition performance in trials where the sets presented were 2 versus 3, and 3 versus 4, respectively. Error bars denote confidence intervals (95%).

For this analysis, we split the dataset and only used 2-knowers. The model included cardinal recognition as the dependent variable, with a random intercept for participants, and fixed effects for number combination. The mixed model demonstrated no effect of number combination (*F*_1, 26_ = 1.64, *p* = 0.21), indicating no difference in performance between the number combinations 2 versus 3 and 3 versus 4. This could suggest that 2-knowers could identify both 3 and 4 without needing to contrast it to known number words. However, looking at figure 5, we do see a mean difference between the trials, with a lower mean performance in trials presenting 3 versus 4. It could suggest that we would find a significant difference between the trials if we had greater statistical power.

## 4. Discussion

In this study, we set out to investigate the ability of cardinal recognition within subset knowers. The results suggest that on a group level, all subset knowers (i.e. 1- to 4-knowers) could identify all presented cardinalities at rates above chance, indicating an approximate understanding of numbers beyond their current knower level. The exploratory analyses indicated that there might be an upper limit to this ability though, as 1-knowers seemed to struggle identifying targets 3 and 4. The following discussion will first focus on the group level, discussing what subset knowers seem to understand about number words and cardinality. Next, we discuss the possibility of an upper limit in cardinal recognition. Then, we discuss whether contrast or recognition drives this performance. We conclude with some directions for future research.

First, the results indicate that on a group level, subset-knowers, i.e. 1- to 4-knowers, have some approximate understanding of numbers, consistent with suggestions made by Silver *et al*. ([9]; also see e.g. [8,13]). Specifically, previous research has demonstrated that subset knowers can use known number words to identify unknown number words (among others, see [5,15,32]). For example, when 2-knowers are tasked with discriminating between sets of 2 and 4 in an interaction with an adult, they can generally tell which set is 4 [9,32]. Indeed, the performance of our participants can be interpreted as evidence for that subset-knowers can, at the very least, spontaneously recognize sets of known number words, contrast this to sets of unknown number words, and then use this information to identify the correct set. Thus, they can at least use their current knowledge of numbers to orient towards unknown numbers, possibly aiding in developing their cardinal knowledge further. In turn, this suggests that subset-knowers have understood several basic principles about number words. First, the results provide further evidence that they understand that number words denote a specific cardinality. Further, being able to spontaneously contrast known and unknown sets indicates that they have an exact representation of at least 1, and that they have understood that number words are mutually exclusive—e.g. that ‘one' only refers to a set of 1.

In our mixed model 1, we saw an effect of knower level, and looking at figure 2*c*, it indeed looks like children with higher knower levels (e.g. 4-knower) performed better at the task, compared to those of a lower knower level (e.g. 1-knower). This is indeed expected. However, the following post-hoc comparisons, descriptive statistics, and the exploratory mixed model 1.1 and 1.2 indicated interesting differences between pre-, 1- and 2-knowers (and above). It tentatively seems that 1-knowers perform at rates above chance when the target was 1 and 2, but struggled to identify the target when it was 3 or 4—indeed, this performance was on a similar level to pre-knowers. At the same time, 2-knowers seemed to identify all presented targets at rates above chance. A potential explanation for this is that subset-knowers have an approximate understanding of numbers that is ‘one more' their knower level (in line with [8,13]). That is, it could be that 1-knowers have an approximate understanding of 2, such that they can recognize that set, but not of 3 or higher. Similarly, it could explain why 2-knowers can identify all targets above chance; if they have an approximate understanding of 3, and can thus recognize that set, it should be relatively easy to identify 4—as this is the only completely unknown number left. As such, these results might lend further support to the notion that subset knowers do recognize sets above their current knower level, but that this recognition has an upper limit that might be expressed as knower level + 1 [13]. However, as we did not observe any significant difference between pre- and 1-knowers, nor 1- and 2-knower performance, we must take this interpretation with caution, and we cannot say that this potential difference, or explanation, is true. Indeed, it is also possible that there are differences between pre- and 1-knowers across all targets, and/or that there are no actual differences between 1- and 2-knower performance. Still, we argue that at the very least, this observed pattern is an exciting venture that warrants further exploration in future studies.

If the proposed knower level + 1 hypothesis is true, it would also suggest that subset-knowers spontaneously recognize sets in this task, rather than spontaneously contrast known number words to unknown number words. However, another potential explanation for the (descriptive) difference between 1- and 2-knowers might also be that 2-knowers have an exact understanding of both 1 and 2, meaning that they had more trials in which they could contrast known number words to unknown number words. As to discern which explanation is more likely, we conducted the exploratory mixed model 2. Here, we observed no significant difference in 2-knower performance in the number combinations of 2 versus 3 and 3 versus 4.

At face value, this result might indicate that 2-knowers can find the correct target in both combinations, in turn suggesting that they spontaneously recognize given sets. However, looking at figure 5, we can see a descriptive mean difference between the combinations, where it looks like 2-knowers performed somewhat worse with the combination 3 versus 4. In turn, this might suggest that 2-knowers performed worse when there was no known number word to contrast to. That we cannot find a significant difference here might be due to statistical power; with more trials and/or more participants, we might have observed a significant difference. On the other hand, it is also theoretically possible there is no true difference here. Therefore, it is still quite possible that 2-knowers identify the correct targets by contrasting known number words with unknown number words, but it is also possible that they spontaneously recognize given sets.

Indeed, one could also argue that the design of the task made contrasting difficult. The participants were presented with a voice which called attention to the stimuli, and then asked for a target cardinality; e.g. ‘Look, cars! Can you find three?'—taking 5 s. Thus, participants needed to process auditory and visual information at the same time during the prompt. Then, we recorded first gaze within 200 ms after the prompt had ended. Thus, to contrast the sets against each other, the 2-knowers would first have to attend to the voice and prompt while simultaneously processing the known cardinality (e.g. there is 2 on the left side). Then they would have to process the target word, e.g. ‘three', and contrast this to the known cardinality and dismiss this set (e.g. ‘there is 2 on the left side, which is not 3, so it cannot be the left group'), and then choose the correct target (if not on the left side, it must be on the right side)—all within 200 ms. They would also have needed to perform this contrast without looking at the incorrect set first, which in this case would have been their reference point. Considering the age of our participants, it could be argued that this is a very complex contrast being made in a very short time, especially given that the reaction times of saccades on average are around 200 ms at this age [29]. However, more research is needed to give a more definitive answer to this question.

Indeed, it is still unclear how 1- and 2-knowers solved the eye-tracking task, and how their performance might be limited. If they recognize given sets, it would lend further support to the notion that subset-knowers have an approximate understanding of numbers before they produce cardinality themselves. Further, it could be that this approximate understanding can be expressed as knower level + 1, and that such an understanding might very well be the transitional state between knower levels (e.g. [8]). If children instead contrast their known sets to unknown sets, it would suggest that children use their current exact representations of sets to identify and explore new, unknown sets. To be able to discern between these possibilities, and to explore the knower level + 1 hypothesis further, we argue that future research should focus on four steps. First, it would be beneficial to include a larger group of 1- and 2-knowers, as to be able to analyse their performance in even more depth. Second, it is important to include more trials with both known and unknown number words. This would make a comparison between these combinations more informative. Third, it might also be interesting to present larger numbers as well. If the knower level + 1 hypothesis is correct, 2-knowers should not be able to recognize unknown large sets, e.g. 4 versus 6. Fourth, we propose the use of the ‘fast cards' task, or a similar task. In this task, children are presented with a single given set, and are tasked with identifying how many objects there are [33]. Comparing the performance in such a task with the current eye-tracking task could provide further insight into whether children can recognize sets, without the need of a contrasting set.

Before concluding, we also need to address a methodological choice made here. The way we implemented the give-n task might make it difficult to differentiate between 4-knowers and CP-knowers—since the highest asked for number was 4. However, as the model we used to derive knower levels weighs in prior and posterior likelihood of certain responses, it is quite sensitive for each trial response. For example, it means that if a child responded incorrectly to e.g. ‘four' one time (but correct in all other trials), the probability of being a CP-knower decreased, while being a 4-knower increased. Thus, based on the probability of responses, the model can differentiate between 4-knowers from CP-knowers with only four items [34]. Even so, we cannot exclude the possibility that some participants were misclassified as either a 4-knower or CP-knower. We note that this is always a risk when using the give-n task [34], and is not unique for this study, and importantly, that it does not affect our conclusions. The main aim of this paper was to investigate what subset knowers, and especially 1-knowers, understand about number words. The inclusion of CP-knowers was mainly to ascertain that the eye-tracking task worked as intended.

Further, a limitation of this study is the relatively high amount of missing data in our eye-tracking task. Indeed, the highest number of valid trials was 65 (out of 72), so no child contributed with full data. Even though relatively high attrition rates are common in studies with young children (e.g. [28]), this might suggest that the task was too taxing for children in this age range. Indeed, we presented both category trials and cardinal trials, and participants had 5 s to look at the target after each prompt. These choices were made so as to ensure task integrity. Even so, it is possible that this caused the stimuli presentation to be too long for these young participants (and perhaps the category trials were too easy and thus under-stimulating). We suggest that future research should only present cardinal trials, and have a shorter time window after the prompt ends, as to hopefully make the task more engaging and less taxing.

In sum, we demonstrate that subset knowers, from 1-knower and up, can at the very least spontaneously contrast known sets to unknown sets, and then use this information to find cardinal targets. In turn, this further strengthens the notion that the understanding of number words begins at the 1-knower level. It is possible that this performance can be explained by an approximate understanding of numbers, but future research is needed to better understand how subset-knowers solve this task.

## Data Availability

All data, analysis and code are available for reviewers at OSF; https://osf.io/k3bzq/?view_only=ee8417ffe794478886bccf96f64f4d38. You will have access to the raw eye-tracking data, and the analysis work flow for creating workable datasets (the Timestudio .study file). You will also find the code used for creating the data matrix used in the article (SPSS syntax files), for both sample 1, 2 and the merged sample 1 and 2 dataset. Finally, see the jamovi files for detail about the main analysis model, such as structure of the models, and controls being made. Also note that the supplementary material presented contain additional information on tasks, analyses and data. The data are provided in electronic supplementary material [35].

## Footnotes

1 The model did not fulfill the mixed model assumptions (see electronic supplementary material). However, Schielzeth *et al*. [31] demonstrated that mixed models are robust against assumption violations. Furthermore, a secondary mixed model with proportional looking time as the dependent variable demonstrated similar results (see electronic supplementary material). Together, these findings suggest that the results are reliable. As such, we continued with post-hoc analyses.
